# N^6^-Adenosine Methylation in RNA and a Reduced m_3_G/TMG Level in Non-Coding RNAs Appear at Microirradiation-Induced DNA Lesions

**DOI:** 10.3390/cells9020360

**Published:** 2020-02-04

**Authors:** Alena Svobodová Kovaříková, Lenka Stixová, Aleš Kovařík, Denisa Komůrková, Soňa Legartová, Paolo Fagherazzi, Eva Bártová

**Affiliations:** 1 Institute of Biophysics of the Czech Academy of Sciences, Královopolská 135, 612 00 Brno, Czech Republic; aluskakovarikova@centrum.cz (A.S.K.); lenka@ibp.cz (L.S.); kovarik@ibp.cz (A.K.); komurkova@ibp.cz (D.K.); legartova@ibp.cz (S.L.); fagher@ibp.cz (P.F.); 2 Department of Experimental Biology, Faculty of Science, Masaryk University, Kamenice 753/5, 625 00 Brno, Czech Republic

**Keywords:** DNA repair, RNA methylation, METTL-like enzymes, histones, epigenetics

## Abstract

The DNA damage response is mediated by both DNA repair proteins and epigenetic markers. Here, we observe that N^6^-methyladenosine (m^6^A), a mark of the epitranscriptome, was common in RNAs accumulated at UV-damaged chromatin; however, inhibitors of RNA polymerases I and II did not affect the m^6^A RNA level at the irradiated genomic regions. After genome injury, m^6^A RNAs either diffused to the damaged chromatin or appeared at the lesions enzymatically. DNA damage did not change the levels of METTL3 and METTL14 methyltransferases. In a subset of irradiated cells, only the METTL16 enzyme, responsible for m^6^A in non-coding RNAs as well as for splicing regulation, was recruited to microirradiated sites. Importantly, the levels of the studied splicing factors were not changed by UVA light. Overall, if the appearance of m^6^A RNAs at DNA lesions is regulated enzymatically, this process must be mediated via the coregulatory function of METTL-like enzymes. This event is additionally accompanied by radiation-induced depletion of 2,2,7-methylguanosine (m_3_G/TMG) in RNA. Moreover, UV-irradiation also decreases the global cellular level of N^1^-*methyladenosine* (m^1^A) in RNAs. Based on these results, we prefer a model in which m^6^A RNAs rapidly respond to radiation-induced stress and diffuse to the damaged sites. The level of both (m^1^A) RNAs and m_3_G/TMG in RNAs is reduced as a consequence of DNA damage, recognized by the nucleotide excision repair mechanism.

## 1. Introduction

Focusing on epigenetic features, it has been established that DNA methylation, posttranscriptional modifications of RNAs, and posttranslational modifications (PTMs) of histones are essential for genome functions. For example, DNA modifications that regulate gene expression include the methylation (5mC) or hydroxymethylation of cytosine (5hmC). On the RNA level, a functionally significant role is attributed to 5-methylcytidine (m^5^C), 5-hydroxymethylcytidine (hm^5^C), N4-acetylcytidine (ac^4^C), 2,2,7-methylguanosine (m_3_G/TMG), N^1^-*methyladenosine* (m^1^A), and/or N^6^-methyladenosine (m^6^A) [1,2,3,4,5,6,7,8]. It is well known that the N^6^-methyladenosine (m^6^A) sites in eukaryotic mRNA have a very significant regulatory role. It has been found that m^6^A RNA occupies the 3′-untranslated regions (3′-UTRs) and also is located near the stop codon of the mRNA [9,10,11]. To some extent, this posttranscriptional modification affects pre-mRNA splicing, RNA degradation, and specific protein–RNA interactions [5,12,13,14]. Importantly, m^6^A can also appear in transfer RNA (tRNA), ribosomal RNA (rRNA), and/or long-noncoding RNAs (lncRNAs) [15]. This epigenetic mark is catalyzed by methyltransferases METTL3 (methyltransferase-like 3) and METTL14 (methyltransferase-like 14) [12,16,17]. The METTL14 enzyme is considered catalytically inactive and is the preferred binding partner for the enzymatic activity of the METTL3 protein [2,18]. Importantly, the function of METTL3 was found to be changed in several tumor cells. For example, Dahal et al. showed that METTL3 is upregulated in melanoma, and depleting METTL3 appeared in parallel with a reduced level of m^6^A RNAs and diminished melanoma invasiveness [19]. Methylation of N^6^-adenosine in RNA is also mediated by the methyltransferase-like 16 (METTL16) protein, which is responsible for generating m^6^A in U6 small nuclear RNA (snRNA). Moreover, METTL16-mediated modification at the 5′ splice sites is considered as an important component of the splicing machinery [20]. Moreover, Doxtader et al. showed that METTL16 regulates a key metabolite of homeostasis, S-adenosylmethionine SAM, which is a well-known DNA methylation cofactor [21].

Xiang et al. also showed that m^6^A RNAs participate in the DNA damage response (DDR) in cells exposed to UV light. Due to the fact that methylated RNAs are found at UV-damaged chromatin, it makes sense that the regulatory METTL3 enzyme should also be recruited to UV-damaged chromatin. On the other hand, knocking out METTL3 did not affect the recruitment of crucial DNA repair-related factors, including XPA, 53BP1, and BRCA1, to microirradiated chromatin. Extensive UV-induced recruitment of METTL3 to DNA lesions was found to be dependent on the function of *Poly (ADP-ribose) polymerase 1* (PARP1), as shown by the PARP inhibitor abrogating METTL3 accumulation in UV-damaged chromatin [22]. Xiang et al. also observed that METTL3 and METTL14 act in parallel with Polymerase κ (Pol κ), playing a role in several DNA repair pathways. In this regard, Pol κ colocalizes with m^6^A RNAs to the damaged chromatin that is dense in cyclobutane pyrimidine dimers (CPDs). Therefore, m^6^A RNAs likely regulate the nucleotide excision repair (NER) mechanism [22,23]. The primary function of NER is the elimination of DNA adducts appearing as a consequence of UV irradiation or the cell treatment by cytotoxic drugs, including those used as a cytostatic treatment. In general, the NER pathway is mediated via two mechanisms: global genome NER (GG-NER) or transcription-coupled NER (TC-NER) [24,25]. GG-NER recognizes DNA lesions irrespectively of the type of chromatin, while TC-NER works in the damaged transcribed strand of active genes. GG-NER is mediated via repair factors, including XPC-RAD23B. On the other hand, TC-NER is activated by RNA polymerases stalled at lesions. Moreover, proteins, like CSA, CSB, and XAB2, contribute to this process [26,27,28].

The dynamic behavior of m^6^A RNAs is regulated not only by specific “writers” (adenosine methyltransferases METTL3, METTL14, and METTL16, but also by “erasers” (m^6^A RNA demethylating enzymes) [5]. For instance, m^6^A RNA demethylation is mediated by ALKBH5 demethylase [12,16,17,29,30,31,32,33,34]. FTO (an obesity-associated factor) is also a highly specific m^6^A RNA “eraser” [35]. Several studies have shown that FTO regulates the function of cancer stem cells, cancer cell growth, and self-renewal via the demethylation of m^6^A RNAs [36]. Interestingly, FTO is transiently recruited to damaged, γH2AX-positive chromatin. Recruitment of FTO to DNA lesions is relatively fast, being completed 4-10 min after irradiation [22]. Interestingly, this demethylase also colocalizes, to some extent, with nuclear speckles; thus, FTO may also contribute to the splicing process [37,38]. However, it remains unclear how splicing is affected in the case of genome injury. In this regard, Legartová et al. identified a high density of the DNA repair protein 53BP1 in nuclear speckles, enriched in pre-mRNA splicing factors [39]. This discovery aligns well with FTO having a regulatory function in both alternative splicing and DNA damage repair [22,40].

Based on the above-mentioned data, we studied the extent to which methylation of RNAs and its functions are changed when DNA damage is induced by UV light. Moreover, we analyzed if seemingly unrelated depletion of histone H3 trimethylation at the lysine 9 position affects RNA methylation at UV-damaged chromatin. Along with others [22], we also investigated the kinetics of RNA recruitment and RNA processing in UVA-induced CPD sites. We preferentially studied the role of m^6^A RNA at the UV-irradiated genomic region, and we also focused on the DNA damage-related function of additional reversible modifications of RNAs, including 2,2,7-methylguanosine (m_3_G/TMG) and N^1^-*methyladenosine* (m^1^A). In the DNA repair process, we also analyzed how selected splicing factors are affected by local laser microirradiation, inducing the NER pathway. Together, here we specify the NER mechanism from the view of several features, specifically for the epitranscriptome and splicing machinery.

## 2. Materials and Methods

### 2.1. Cell Cultivation and Treatment

Wild-type wt and Suv39h1/h2 double-knockout dn mouse embryonic fibroblasts (MEFs) were cultivated in Dulbecco’s modified Eagle’s medium supplemented with 10% fetal calf serum (FCS) (Merck, Darmstadt, Germany). The medium was supplemented with 1 μL of β-mercaptoethanol (#31350-010, Thermo Fisher Scientific, Waltham, Massachusetts, USA), 5 mL of nonessential amino acids (100×; #1140-035, Thermo Fisher Scientific), 5 mL of sodium pyruvate (#11360-039, Thermo Fisher Scientific), and 1.5 g of NaHCO_3_.

For the analysis of the impact of H4K20 methylation (essential for non-homologous end-joining repair; NHEJ) on m^6^A RNA recruitment to DNA lesions, we used compound A-196 (#SML1565, Sigma Aldrich, St. Louis, MO, USA). This epi-drug is considered a selective chemical inhibitor of the SUV4-20H1 and SUV4-20H2 histone methyltransferases (HMTs) responsible for H4K20me2/me3 [41]. In these experiments, we used a final concentration of 5 μM of this inhibitory drug (information for a specific concentration, see [42]).

To inhibit RNA polymerase I and RNA polymerase II, cells were treated by actinomycin D (0.5 µg/mL) for 2 h and by α-amanitin (2 µg/mL) for 2 or 4 h. After the treatment, cells were washed twice by PBS, a new cultivation medium was added, and after that, microirradiation experiments were performed.

The cells cultivated on 50-mm glass-bottom dishes (No. 0; MatTek Corporation, Ashland, MA, USA, #P50G-0-30-F) were also irradiated with a UVA lamp (model GESP-15, 15 W, UVA 330–400 nm wavelength with maximum efficiency at 365 nm) and UVC lamp (Philips, Amsterdam, The Netherlands, model TUV 30 W T8, UVC 254 nm wavelength). The cells were irradiated by the UVA and UVC lamps for 10 min, and then the cells were fixed at multiple intervals (2 min, 3 h, and 24 h after irradiation). The lamp distance from the sample was 2 cm for the UVA source and 70 cm for the UVC source [43]. The cells were also irradiated by 5Gy of γ-rays (Co-60 was a source of γ-radiation).

### 2.2. Whole-Genome Bisulfite Sequencing and Cytosine Methylation Analysis

We sequenced DNA from Suv39h1/h2 wt and Suv39h1/h2 dn cell lines using Whole Genome Bisulfite Sequencing (WGBS) technology from the BGI company (Hongkong, China) [44]. For the WGBS library construction, the DNA was fragmented by sonication using a Bioruptor (Diagenode, Belgium) to a mean size of approximately 250 bp, followed by blunt-ending and dA addition to the 3’-end. Finally, adaptor ligation was performed (in this case, methylated adaptors were used to protect against bisulfite conversion). The procedure was performed according to the manufacturer’s instructions. Ligated DNA was bisulfite converted using the EZ DNA Methylation-Gold kit (ZYMO, Irvine, CA, USA). Insert fragments of different sizes were excised from the same lane of a 2% TAE agarose gel. Products were purified using a QIAquick Gel Extraction kit (Qiagen, Hilden, Germany) and amplified by PCR. Sequencing was performed using the High Seq Illumina 4000 sequencer generating approximately 127 Gb data, which corresponded to a mouse genome coverage of 50%. After removal of low quality reads (Phred score <20 and over >10% read length), N reads and adaptor sequence clean reads (>1.1 billion) were mapped to the Mus musculus annotated genome (Bioproject: PRJNA20689) using BSMAP [45]. Only reads that mapped uniquely to the reference genome were considered for the methylation analysis. Therefore, 93.4% of clean Suv39h/h1/h1 wt and 92.9% of Suv39h1/h2 dn reads were mapped to the reference. Methyl cytosine identification was performed according to the method and correction algorithm of [46]. Only the methylated cytosines (mCs) covered by at least four reads were used to calculate the methylation level. The degree of bisulfite conversion was estimated by internal phage lambda DNA control and found to be >99%. The methylation level was determined by dividing the number of reads covering each mC by the total reads covering that cytosine according to the formula Nm/(Nm + Nu) × 100%. Nm represents the number of mC reads, while Nu represents the number of nonmethylation reads. Documentation of the output was carried out with the aid of MS Excel and R-studio programs.

### 2.3. Immunofluorescence Staining

Immunofluorescence was performed following [47]. The cells were fixed in 4% paraformaldehyde (PFA) for 10 min at room temperature (RT), permeabilized with 0.2% Triton X-100 (Merck) for 15 min (Merck), and washed twice in phosphate-buffered saline (PBS) for 10 min. Bovine serum albumin (Merck) (1% dissolved in PBS-Tween 20 (0.1%)) was used as a blocking solution. The following antibodies were used at a 1:100 dilution: m^6^A (#202 111, SYSY Antibodies, Goettingen, Germany), anti-FTO (#ab92821, Abcam, Cambridge, UK), anti-fibrillarin (#ab4566, Abcam), anti-METTL3 (#A8370, Abclonal, Woburn, MA, USA), anti-METTL14 (#A8530, Abclonal), and anti-METTL16 (#HPA020352, Atlas Antibodies, Bromma, Sweden). Additionally, we used anti-2,2,7-trimethylguanosine (m_3_G/TMG) (#RN019M, Ribonomics, MBL, Nagoya, Japan) to study m_3_G/TMG RNA. We also analyzed splicing factor SC35 using anti-SC35 (ab#11826, Abcam) and NOCL1 (#A5899, Abclonal), the SMD1 protein (#LS-C346290, 151043, LSBio, Seattle, WA, USA) and N^1^-methyladenosine (m1A) mouse monoclonal antibody (#D345-3MBL); we also used anti-CPDs (#NMDND001, Cosmo Bio., Ltd., Tokyo, Japan) in order to study the NER pathway. The dilution of the primary antibody against the CPDs was 1:100 (#CAC-NM-DND-001, Cosmo Bio).

The following secondary antibodies were used: Alexa 488-conjugated goat anti-rabbit (#ab150077, Abcam), Alexa 594-conjugated goat anti-rabbit (#A11037, ThermoFisher Scientific), Alexa 488-conjugated goat anti-mouse (#A11029, ThermoFisher Scientific), and Alexa Fluor^®^ 594 anti-mouse (#A-11032, ThermoFisher Scientific). As a negative control, we used samples incubated without primary antibodies. Instead, we used 1% BSA dissolved in PBS-Tween. For visualization of cell nuclei, we used 4′,6-diamidino-2-phenylindole (DAPI; Merck) dissolved in Vectashield (Vector Laboratories, Burlingame, CA, USA).

### 2.4. Western Blotting

Western blotting was performed using the methods reported by [47]. We used the following primary antibodies: anti-phosphorylated histone H2AX (γH2AX; phospho S139; #ab2893, Abcam), anti-FTO (#ab92821, Abcam), anti-α-tubulin (#ab80779 Abcam), anti-DNMT1 (#ab188453 Abcam), anti-H3 (#ab7091, Abcam), anti-H3K9me3 (#ab8898, Abcam), anti-METTL3 (#A8370, Abclonal), anti-METTL14 (#A8530, Abclonal), and anti-METTL16 (#HPA020352, Atlas Antibodies). As secondary antibodies, we used anti-rabbit IgG (#A-4914, Merck, Germany; dilution 1:2000), anti-mouse IgG (#A-9044, Merck; dilution 1:2000), and anti-mouse IgG1 (#sc-2060, Santa Cruz Biotechnology, Dallas, TX, USA; dilution 1:1000). By the use of Western blots, we performed analysis of 3 biological replicates.

### 2.5. Laser Scanning Confocal Microscopy, Image Analysis and Induction of DNA Lesions by Local Microirradiation

Images were acquired by laser scanning confocal microscopy. We used a TCS SP5-X confocal microscope system and/or a Leica SP-8 confocal microscope (Leica, Wetzlar, Germany). For observation and image acquisition, we used a 63× oil objective (HCX PL APO, lambda blue) with a numerical aperture (NA) = 1.4. For image acquisition and analysis, the following lasers were used: white light laser (WLL; wavelengths of 470–670 nm in 1-nm increments) and UVA lasers (355-nm and 405-nm). Cells were seeded on microscope dishes (#81166, Ibidi, Fitchburg, WI, USA) until they reached 70% confluence. The cells were maintained under optimal cultivation conditions in an incubation chamber (EMBL) at 37 °C, and the cell culture hood was supplemented with 5% CO_2_. For local laser microirradiation, we selected a region of interest (ROI), and the irradiated area in the genome was approximately 2 µm^2^. Microirradiation was performed by both lasers (355-nm and 405-nm of the wavelength). The lasers were connected to a Leica SP5 X confocal microscope (see description in [47]). In the case of the SP8 confocal system, we used a 405-nm laser line. LEICA LAS AF software was used for image acquisition and analysis. In the locally microirradiated ROIs, we studied the level of the following epigenetic markers as well as markers of DNA damage: m^6^A RNAs, METTL3, METTL14, METTL16, FTO, γH2AX, UBF1/2, fibrillarin, CPDs, m_3_G/TMG RNAs, and m^1^A RNA. Experiments were performed in living and fixed Suv39h1/h2 wt and Suv39h1/h2 dn cells and in HeLa, U2OS, and HaCaT cells. After the immunostaining procedure, locally microirradiated cells were detected according to the registered coordinates on the gridded microscope dishes. Image acquisition was performed at a resolution of 1024 × 1024 pixels at 400 Hz frequency, and we used a bidirectional mode of scanning at 64 lines with a zoom >8×. For the data analysis, we monitored 50–60 cell nuclei, and we performed an analysis of 3 biological replicates. From hundreds of analyses, we know that DNA lesions, induced by 355-nm UVA laser, are CPD- and γH2AX-positive, but cells microirradiated by 405-mn laser are absent of CPDs; thus, it was not essential to perform dual-immunostaining in all the cases studied.

### 2.6. STED Microscopy

An inverted microscope DMI6000 AFC Bino with a laser scanning confocal head Leica TCS SP8 was used for the 3D-STED measurement. This microscope system is equipped with a motorized stage with Super Z-Galvo scanning insert and STED White Objective CS 100×/1.40 OIL. The confocal head consists of two fluorescence PMT detectors and two highly sensitive HyD detectors with a time-resolved gating function. The system was also fitted with a STED 3X module with 660-nm and 775-nm depletion lasers, respectively, which enabled super-resolution imaging by stimulated depletion of emission (up to 50-nm lateral, 130-nm axial resolution). The image acquisition was performed using a depletion laser with a 775-nm wavelength and 3X 3D STED, with gating of 0.2–6.0 ns for N^6^-methyladenosine conjugated with an Abberior STAR 580 secondary antibody. The acquired image z-stacks were deconvolved using Huygens Professional software.

### 2.7. Statistical Analyses and Quantification of the Fluorescence Intensity

The density values for the Western blot fragments and immunofluorescence signals were analyzed by ImageJ (NIH freeware, Bethesda, MD, USA). We also measured the relative fluorescence intensity in the microirradiated ROI. The ROI data were normalized, and the fluorescence intensity measured outside the microirradiated regions. Scanning of the Western blot fragments on fluorescent gels was performed by a GE Typhoon FLA 9000 gel imager and ImageQuant TL 8.2 software (GE Healthcare, Uppsala, Sweden). The collected data from the Western blots were normalized to standards, including the level of the total histone H3 or α-tubulin. Sigma Plot software was used for the statistical analysis by Student’s *t*-test. In each experimental event, we analyzed 50–80 cell nuclei in three independent experiments. We used an online tool http://shiny.chemgrid.org/boxplotr/ for data plotting.

## 3. Results

### 3.1. Reduced DNA Methylation Was Observed in Parallel with a Decrease in H3K9 Trimethylation

**Figure 1:**
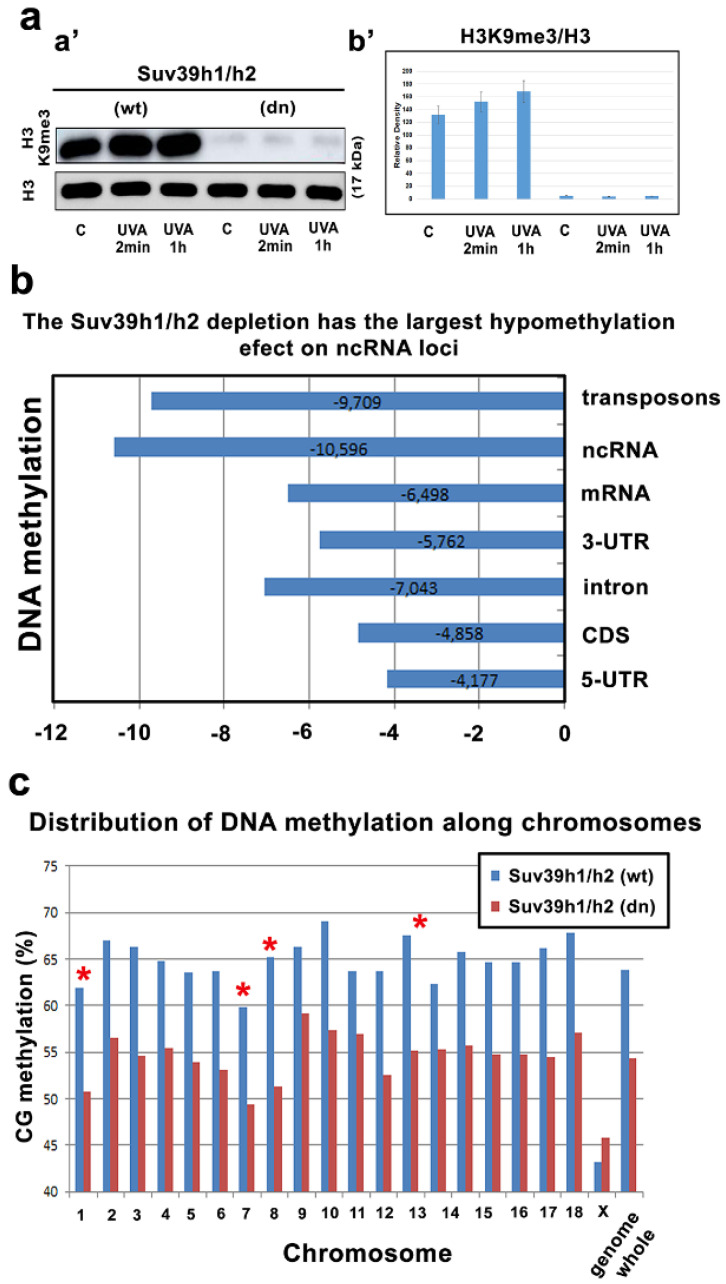
Suv39h1/h2 depleted cells are characterized by global DNA hypomethylation. (**a**,**a’**) H3K9me3 is decreased in Suv39h1/h2 dn cells compared to the level in Suv39h1/h2 wt cells. (**b’**) Data from Western blot analysis were quantified by ImageJ software and normalized to the total protein levels and histone H3. (**b**) DNA methylation in distinct genomic regions was analyzed by whole-genome sequencing. The level of DNA methylation was studied in the Suv39h1/h2 wt and Suv39h1/h2 dn cells. (**c**) Global level of DNA methylation in individual autosomes and chromosome X in the Suv39h1/h2 wt and Suv39h1/h2 dn cells. Red stars show those chromosomes prone to DNA demethylation in Suv39h1/h2-depleted cells. Asterisks (*) show chromosomes characterized by the highest level of demethylation in Suv39h1/h2 depleted cells.

We verified the 5mC level in the DNA of the UV-irradiated Suv39h1/h2 wild-type (wt) cells and Suv39h1/h2-double-knockout (dn) mouse embryonic fibroblasts (MEFs). Our aim was to analyze how potential changes in DNA methylation and depletion of H3K9me3 affect the recruitment of m^6^A RNA to locally induced DNA lesions. Thus, global DNA demethylation, revealed in Suv39h1/h2 double knockout (dn) cells, was an excellent model for these DNA repair studies (Figure 1a–c). As a first, we confirmed that in Suv39h1/h2 dn MEFs, H3K9 trimethylation (me3) is reduced [48,49] (Figure 1a). Moreover, by genome-wide sequencing, we observed DNA demethylation in transposons, noncoding genomic regions, DNA encoding mRNA, and 3-UTRs, as well as in intron regions. The most notable DNA demethylation in Suv39h1/h2 dn cells was found in transposons and noncoding DNA (Figure 1b).

Interestingly, the DNA of individual chromosomes was relatively homogeneously covered by methylation (86.6–96.2%). Low frequency reads mapped to Y chromosome sequences were not counted (both the Suv39h1/h2 wt and Suv39h1/h2 dn cell lines were of the XX genotype). In the mutant cell line, the 5-methylcytosine level was reduced by at least 10% in genes, mapped on the autosomes. We observed reduced 5mC in the DNA of most mouse chromosomes (MMUs), except in chromosome X that was slightly hypermethylated in Suv39h1/h2 dn cells when compared with wild-type counterpart. However, mouse chromosome X was generally less methylated in DNA than the autosomes in both Suv39h1/h2 wt and Suv39h1/h2 dn MEFs. Additionally, we observed that some specific autosomes are more prone to DNA demethylation (for example, MMU1, MMU7, MMU8, and MMU13 were significantly demethylated in mutant cells) (Figure 1c).

### 3.2. Genome Instability and DNA Demethylation, Induced by Suv39h1/h2 Deficiency, Was Accompanied by a Weak Accumulation of m^6^A RNAs at UV-Induced DNA Lesions

**Figure 2:**
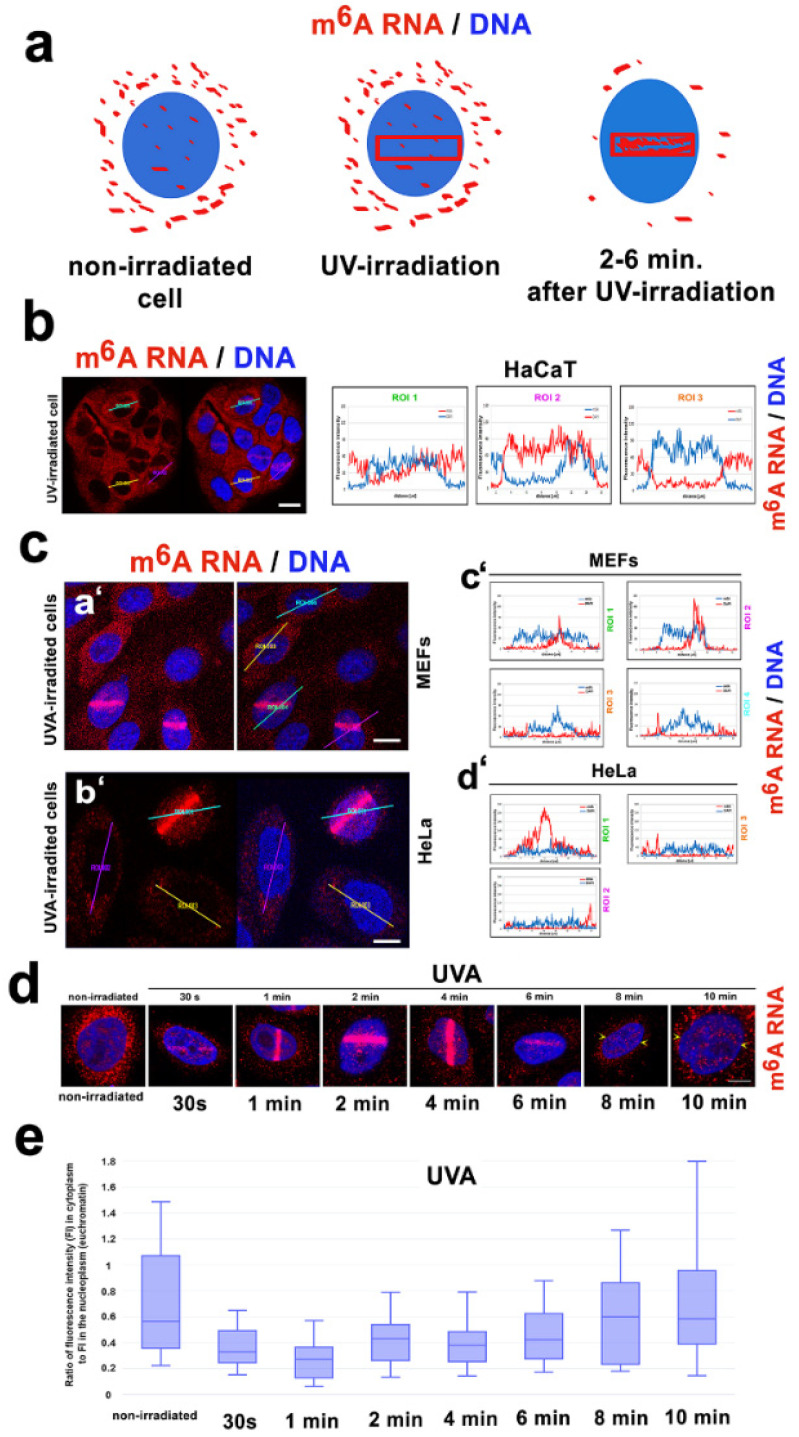
In microirradiated cells, m^6^A RNA diffused from the cytoplasm to the cell nucleus. (**a**) A pictorial illustration of m^6^A RNA distribution in the cytoplasm and the cell nucleus. The red frame in panel (**a**) shows the selected genomic region exposed to local laser microirradiation. (**b**) The distribution of m^6^A RNAs in the human keratinocytes (HaCaT cell line) with physiological genomes. (**c**) m^6^A RNAs in (**a’**), (**c’**) wild-type MEFs and (**b’**), as well as (**d’**) cervical carcinoma HeLa cells. Cells were exposed to laser microirradiation. Quantification of the fluorescence intensity of the Alexa 594-stained m^6^A RNAs was performed using LAS X software. Scale bars represent 15 µm. Diffusion of m^6^A RNA from the cytoplasm to the cell nucleus exposed to (**d**), local laser microirradiation. Scale bars show 5 µm. DAPI (blue) was used as a counterstain. HeLa cells were used for these experiments. Panel (**e**) shows the quantification of the fluorescence intensity in microirradiated ROIs. This analysis was performed by LAS X software connected to a Leica laser-scanning confocal microscope.

We confirmed that m^6^A RNAs accumulate at the microirradiated chromatin (Figure 2a–c) [22]. By immunofluorescence, we found a relatively high level of m^6^A RNAs in the cytoplasm. Specifically, HaCaT keratinocytes were characterized by a higher level of m^6^A RNAs in the cytoplasm compared to the level of m^6^A RNA in the MEFs or HeLa cells (Figure 2a–c). Interestingly, when the cells were microirradiated, the level of m^6^A RNAs in the cytoplasm was rapidly reduced; instead, the m^6^A RNAs accumulated in the microirradiated region inside the cell nucleus (Figure 2(ca’–d’)). Accumulation of m^6^A RNAs in ROIs was 0.5–6 min after laser exposure. This result was additionally verified by time-lapse microscopy, supporting diffusion of m^6^A RNAs from the cytoplasm to the cell nucleus of the microirradiated cells (Figure 2d,e). In detail, non-irradiated cells were characterized by a high level of m^6^A RNAs in the cytoplasm. After microirradiation, m^6^A RNAs appear in irradiated ROI, with the highest level 2–4 min after UVA-laser exposure. At 8–10 min after UVA-irradiation, m^6^A RNAs disappeared from the irradiated regions in the cell nucleus and likely newly methylated RNAs were observed in the cytoplasm (Figure 2d,e).

**Figure 3:**
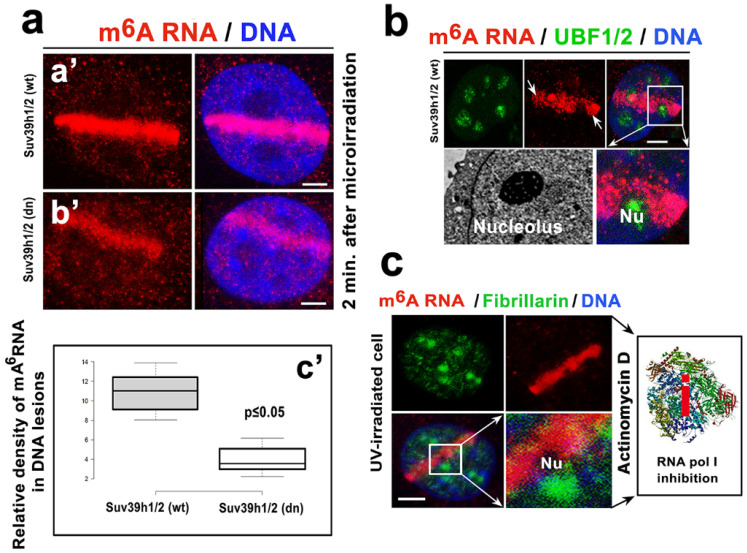
The level of m^6^A RNA was lower in microirradiated Suv39h1/h2-depleted cells and changes in m^6^A RNA were not observed in the nucleoli of cells exposed to UVA light. (**a**) Suv39h1/h2 depletion affected the level of m^6^A RNAs at the locally microirradiated chromatin (compared to the levels in (**a’**) Suv39h1/h2 wt and (**b’**) Suv39h1/h2 dn cells). A relative density of m^6^A RNA in microirradiated ROI is shown in panel (**a**,**c’**). Scale bars in panels a-c represent 5 µm. (**b**) The m^6^A RNAs were absent in the UBF1/2-positive region of the nucleoli, and (**c**) m^6^A RNAs were absent in fibrillarin-positive regions of the nucleoli, in the cells treated by RNA pol I inhibitor, actinomycin D. For a pictorial illustration of RNA pol I inhibition (red i), the structure of the RNA polymerase I at a 2.8 A resolution was taken from the PDB database [50].

**Figure 4:**
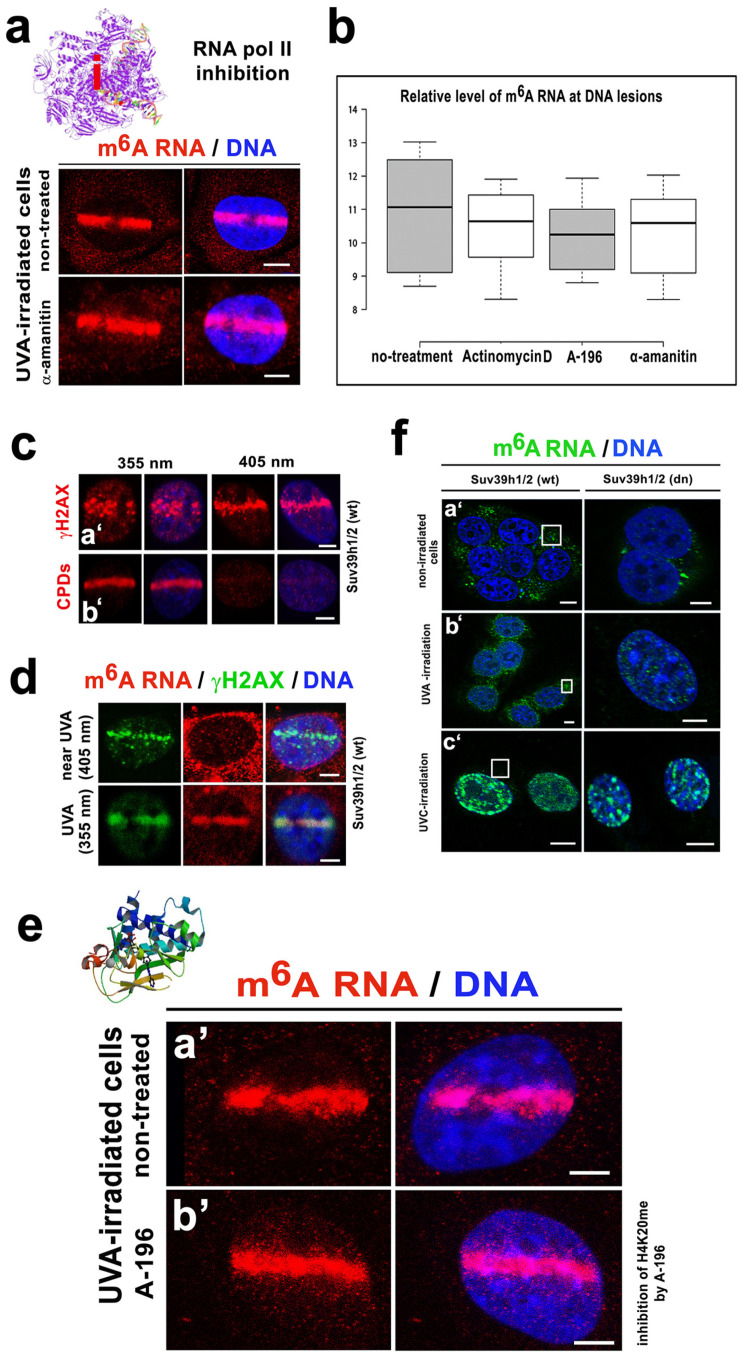
m^6^A RNAs were present at only CPD-positive chromatin, and inhibitors of RNA pol I and RNA pol II did not affect the m^6^A RNA level in the microirradiated regions. (**a**) An inhibitor of RNA pol II, α-amanitin, did not affect m^6^A RNA levels at the DNA lesions. For an illustration of RNA pol II inhibition (red i), the RNA pol II elongation complex was adapted from the PDB database [51]. (**b**) Summary of the inhibitory effects (treatment by actinomycin D, A-196, and α-amanitin) on m^6^A RNA levels. Quantification was performed according to microirradiated ROIs. (**c**) Chromatin with (**a’**) γH2AX positivity in microirradiated ROI and (**b’**) with/without CPDs. Results are shown after exposure to the 355-nm laser or 405-nm laser line. (**d**) Panels show DNA lesions (absent of CPDs) induced by the 405-nm laser line. The 355-nm laser-induced both γH2AX and m^6^A RNAs in the microirradiated genome. (**e**) In comparison to (**a’**) nontreated cells and (**b’**) an inhibitor of H4K20me2/me3, A-196 did not affect the level of m^6^A RNAs in the microirradiated chromatin. A structure of the inhibitor of the SUV4-20h1/h2 methyltransferases, the compound A-196, was adopted from the PDB database (it is an example of H4K20me inhibition) [41]. (**f**) Nuclear distribution of m^6^A RNAs in **a’** nonirradiated, **b’** UVA irradiated, **c’**, and UVC irradiated MEFs (whole-cell cultures were irradiated). White frames show the level of m^6^A RNAs in the cytoplasm. The scale bar in panel (a) represent 8 µm; in panels **c**, **a’**, and **b’** they represent 10 µm; in panels **d**,**e** 5 µm; in panels **fa’**,**b’** bars are 5 µm; and in panels **fc’** it is 10 µm in Suv39h1/h2 wt cells and 15 µm in Suv39h1/h2 dn cells.

Interestingly, the level of accumulated m^6^A RNAs at the UVA-induced DNA lesions was approximately 3-fold lower in the Suv39h1/h2 depleted cells compared with the level of m^6^A RNA accumulation in the Suv39h1/h2 wt cells (Figure 3(aa’–c’)). Additionally, we observed that m^6^A RNAs accumulated at the DNA lesions, with the exception of those induced in the UBF1-positive regions of the nucleoli, where transcription of ribosomal genes proceeds (Figure 3b). Moreover, when we used an inhibitor of RNA pol I (actinomycin D), we did not observe changes in the m^6^A RNA level in microirradiated chromatin (compare Figure 3(aa’,c)). Additionally, the inhibitor of RNA pol II (α-amanitin) did not reduce the m^6^A RNA levels at the microirradiated genome (Figure 4a,b). Based on these results, we suggest that N^6^-methylation of adenosine residues in RNAs that appear in the vicinity of DNA lesions likely concerns noncoding RNAs, but not mRNA or rRNA. This claim is confirmed by the fact that the inhibitors of RNA pol I and RNA pol II did not affect the m^6^A RNA levels at the locally microirradiated chromatin (Figure 3c and Figure 4a).

Here, we confirmed the result of Xiang et al. that m^6^A RNAs accumulate at DNA lesions [22]. Moreover, we found that the inhibitor of RNA polymerase II, α-amanitin does not affect this DNA repair event (Figure 4a,b). We additionally confirmed that this process was a part of the NER mechanism recognizing CPDs (induced by 355-nm UVA laser, but not by 405-nm laser) (Figure 4c,d). To this DNA repair process, we found that the accumulation of m^6^A RNAs at the lesions was not affected by an inhibitor (the chemical compound A-196) of Suv4-20h1/h2 methyltransferases, which is responsible for H4K20me2/me3, recognized by the 53BP1 protein, a component of the NHEJ repair pathway (Figure 4(ea’,b’,b)). These new results support the conclusion of Xiang et al. (2017) showing that m^6^A RNA is not a key component of NHEJ repair, but this post-transcription modification of RNAs is playing a role in the NER mechanism [22].

Next, we also analyzed whether the nuclear distribution pattern of m^6^A RNAs can be changed by genome-wide irradiation. We tested the effect of UVA and UVC irradiation, and we observed that UVC, in particular, had significant potential to increase the level of m^6^A RNAs in the cell nucleus. Specifically, a high dose of UVC caused an accumulation of m^6^A RNA into clearly visible nuclear foci, and the level of m^6^A RNAs was significantly reduced in the cytoplasm (Figure 4(fa’–c’)).

Together these findings suggest that the function of the m^6^A RNAs at the DNA lesions does not likely contribute to 53BP1-H4K20me2/me3-mediated NHEJ repair and is not likely linked to mRNA or rRNA processes. Here, we additionally confirm the conclusions of Xiang et al., showing that m^6^A RNA function is preferentially linked to the NER mechanism [22] (Figure 4c,d).

### 3.3. Coregulatory Function among METTL3, METTL14, and METTL16 May Be Responsible for the Localization of m^6^A RNAs at UVA-Induced DNA Lesions; Alternatively, m^6^A RNAs May Diffuse to DNA Lesions as a Consequence of Cellular Stress

**Figure 5:**
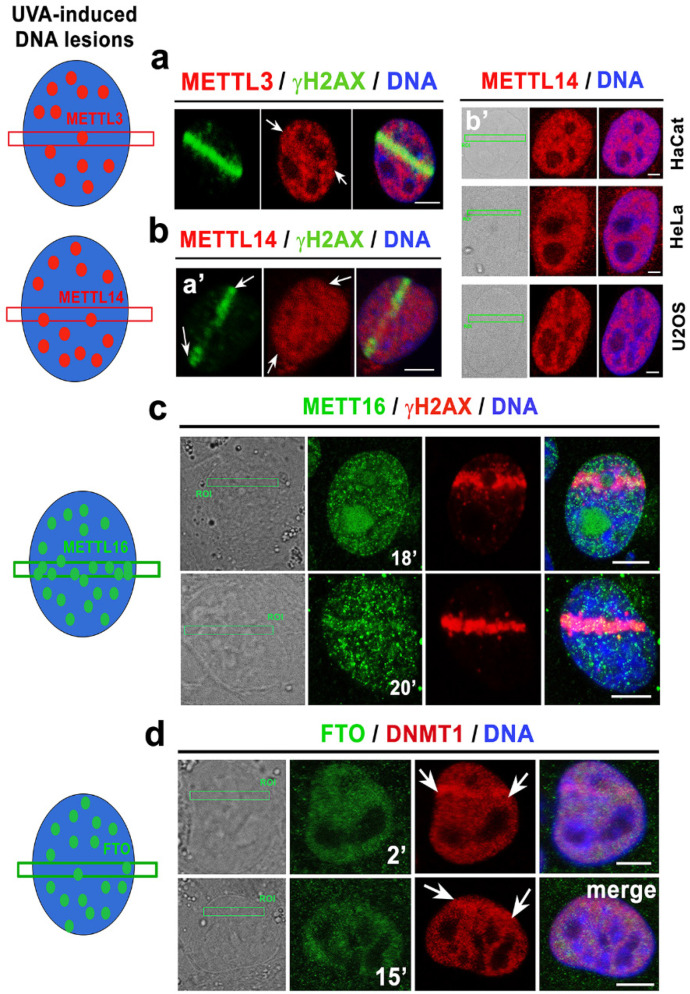
Microirradiation did not affect METTL3/METTL14 and FTO levels, while the METTL16 protein recruits to locally microirradiated chromatin in a subset of irradiated cells. Levels of (**a**) METTL3 in MEF, (**b**) METTL14 in **a’** MEF, and **b’** HaCaT, HeLa, U2OS cells locally irradiated by UVA laser. (**c**) The level of METTL16 in microirradiated MEFs, and (**d**) the distribution pattern of the FTO protein in the microirradiated chromatin of MEFs. The nuclear distribution pattern of METTL3/METTL14 and FTO proteins was not significantly changed by local laser microirradiation, but METTL16 was recruited to microirradiated chromatin in 10% of cells (see lower micrographs in panel (**c**)). Scale bars represent 10 µm.

We observed that UVA microirradiation did not affect the level of the methyltransferases METTL3 and METTL14 at the microirradiated chromatin (Figure 5(a,ba’,b’)). METTL16 (highly dense in both the nucleoplasm and the nucleolus) was relatively stable in the microirradiated genomic regions. However, at later stages of DNA repair, 18–20 min after local laser microirradiation, the METTL16 level was increased in irradiated ROIs. Accumulation of METTL16 to the lesions was observed in approximately 10% of irradiated cells (Figure 5c). An interval of METTL16 accumulation at the damaged chromatin (18–20 min) was not identical to the interval of m^6^A RNA accumulation at DNA lesions (0.5–6 min after UVA-irradiation; compare Figure 3(aa’) with Figure 5c and see Graphical Abstract). Surprisingly, in these intervals, the level of FTO demethylase, antagonizing m^6^A in RNAs, was stable before and after cell exposure to UVA light (Figure 5d).

**Figure 6:**
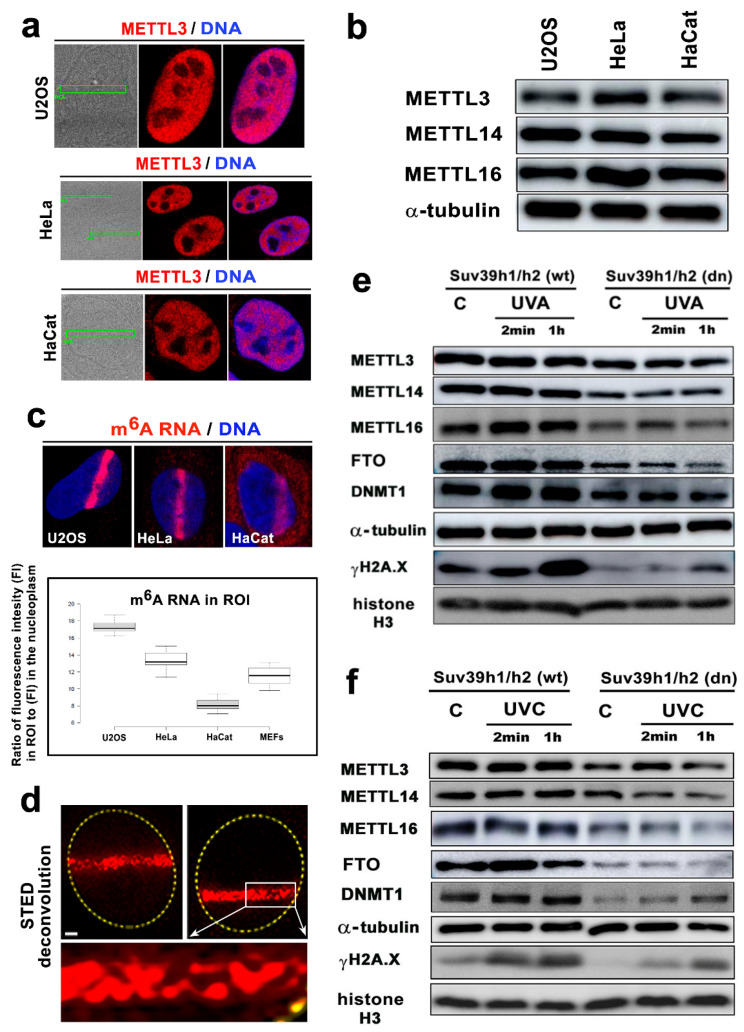
The level of m^6^A RNAs in microirradiated chromatin was the highest in the U2OS osteosarcoma cells. (**a**) METTL3 enzyme was not changed in the microirradiated U2OS and HeLa tumor cells or the HaCaT keratinocytes. (**b**) Western blotting also showed that the levels of METTL3, METTL14, and METTL16 in U2OS, HeLa, and HaCaT cells were almost identical. (**c**) In comparison to the other cell types, U2OS cells were characterized by the most pronounced accumulation of m^6^A RNAs at DNA lesions. In HaCaT cells, the level of m^6^A RNAs at the CPD sites was the lowest compared to that of the other cell types studied (see box plot). Scale bars in panels a-c represent 8 µm. (**d**) STED microscopy showed the focal distribution of the m^6^A RNAs in the microirradiated regions. Scale bars represent 2 µm. Western blot analysis of the levels of METTL3, METTL14, METTL16, FTO, DNMT1, and γH2AX in Suv39h1/h2 wt and Suv39h1/h2 dn nonirradiated cells and cells exposed to (**e**) a UVA lamp and (**f**) a UVC lamp. Protein levels were normalized to the levels of α-tubulin, and the level of histone proteins was normalized to total histone H3.

Additionally, we addressed a question whether METTL3 and METTL14 only accumulate at the microirradiated chromatin in tumor cells in comparison to MEFs (Figure 5(ba’,b’) and Figure 6a). This hypothesis came from the conclusion published by Xiang et al., showing m^6^A RNA accumulation at DNA lesions induced by osteosarcoma U2OS cells and cervical carcinoma HeLa cells [22]. However, also in U2OS and HeLa tumor cells, we found no recruitment of METTL3 and METTL14 enzymes at the DNA lesions.

The same METTL3 profile was detected for the DNA lesions studied in HaCaT keratinocytes (Figure 6a). Interestingly, despite the same levels of METTL3 and METTL14 in the distinct cell types analyzed by immunofluorescence and Western blotting, the U2OS cells were characterized as having the most significant m^6^A RNA accumulation at the microirradiated chromatin, while HaCaT cells had the lowest levels of m^6^A RNA at the microirradiated regions (Figure 6b,c). From a morphological point-of-view, super-resolution STED analysis revealed that m^6^A RNA is arranged into tiny foci appearing in microirradiated chromatin (Figure 6d).

Using Western blotting, we additionally showed that, in comparison to the wild-type cells, the Suv39h1/h2 dn fibroblasts were characterized by lower levels of METTL3, METTL14, METTL16, FTO, and DNMT1. Importantly, in comparison to its non-irradiated counterpart, both UVA- and UVC-irradiation did not affect density studied proteins; as expected, only phosphorylation of histone H2AX (γH2AX), a marker of DNA damage, was increased by irradiation (Figure 6e,f, Supplementary Figure S1).

### 3.4. Irradiation Reduced N^1^-Methyladenosine (m^1^A) in the RNA and Reduced 2,2,7-Methylguanosine (m3G/TMG) Specific for a Cap Structure of Non-Coding RNAs

**Figure 7:**
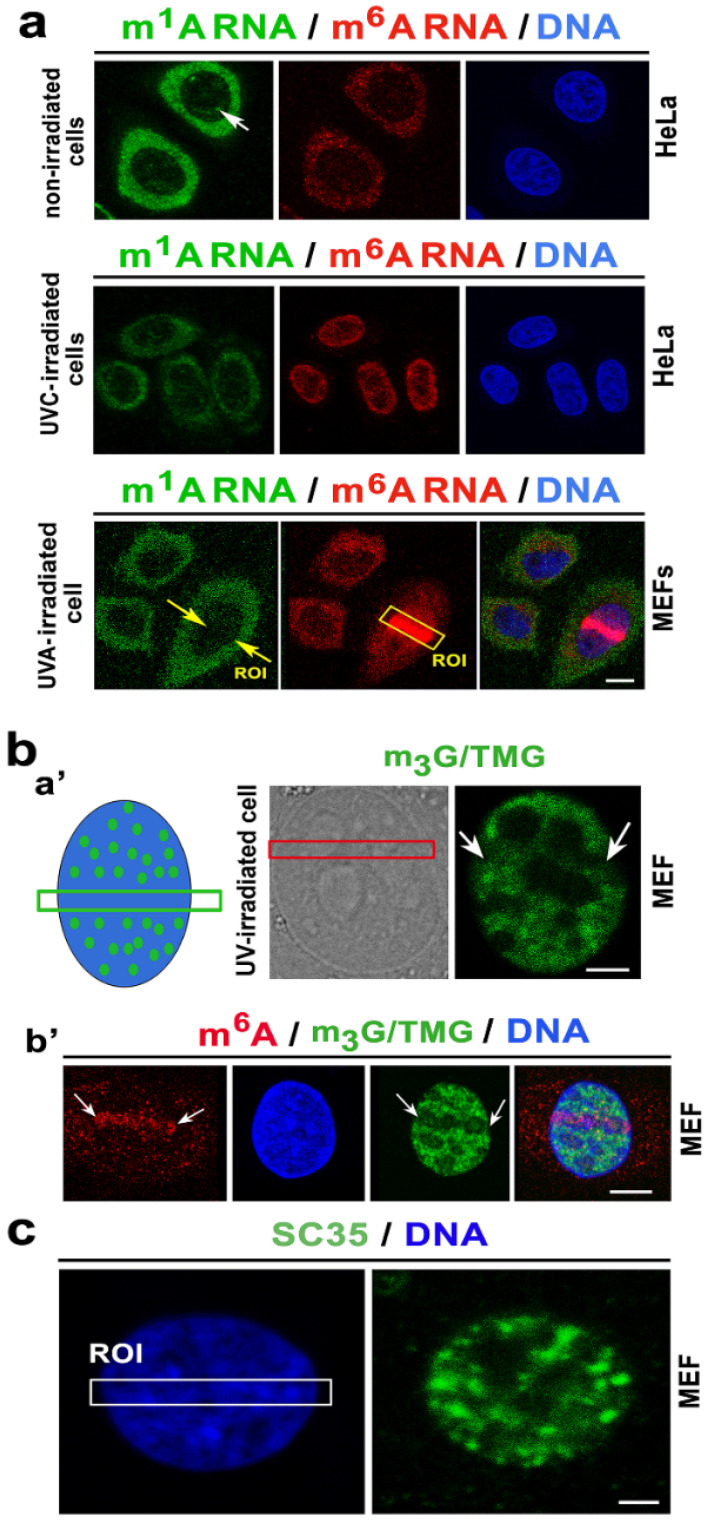
The level of m^1^A RNA is reduced by UV irradiation, and depletion of trimethylguanosine in RNAs (m3G/TMG) appears in the microirradiated chromatin. (**a**) Nuclear distribution pattern of m^1^A RNAs (green) and m^6^A RNAs (red) in nonirradiated cells; globally UVC-irradiated HeLa cells; and MEFs exposed to UVA-microirradiation. The irradiated region of interest is shown as a yellow frame or arrows, labeled as ROI. White arrow shows a tiny focus of m^1^A RNAs inside the cell nucleus. (**b**) The m_3_G/TMG level in (**a’**) UVA-microirradiated MEFs (white arrows and red frame show microirradiated regions); (**b’**) the level of m^6^A RNAs (red) and a reduced level of m_3_G/TMG (green) is shown in UVA-microirradiated MEFs (white arrows indicate the microirradiated genomic regions). (**c**) Local laser microirradiation did not affect the nuclear distribution pattern or the level of splicing protein, SC35. Microirradiated ROI is shown by the white frame. DAPI (blue) was used for visualization of the cell nuclei. Scale bars in panel a represent 10 µm; in panel **ba’**, they represent 5 µm; in panel **bb’**, they represent 10 µm; and in panel **c**, they represent 5 µm.

Here, we additionally studied if other post-transcription modifications of RNAs, in addition to m^6^A RNAs, recognize genomic lesions. We found that N^1^-*methyladenosine* (m^1^A), frequently observed in tRNA and mRNA, is not recruited to DNA lesions. The level of RNAs, abundant on N^1^-methyladenosine (m^1^A), was reduced in the cells exposed to UVC- and UVA-light (Figure 7a). N^1^-methyladenosine (m^1^A) RNAs were found preferentially in the cytoplasm, and only m^1^A-positive tiny foci of RNA were detected inside the cell nucleus of non-irradiated cells. However, we did not observe these foci in the nucleus of UVA- and UVC-irradiated cells. Interestingly, UVC-light caused pronounced m^6^A RNA positivity in the cell nucleus (Figure 7a).

Additionally, the 2,2,7-trimethylguanosine (m_3_G/TMG) level, specific for the cap structure of small RNAs (snRNA, snoRNA), was reduced in the genomic regions exposed to local laser microirradiation (Figure 7(ba’)). Importantly, when the high level of m^6^A RNA, induced by microirradiation, returned back to the normal, the m_3_G/TMG level decreased at the microirradiated chromatin; thus, the specific kinetics of post-transcriptionally modified RNAs were observed at the DNA lesions. These DNA damage-related changes appeared 8–60 min after laser exposure (Figure 2d, Figure 3(aa’), and Figure 7(a,ba’,b’), Graphical Abstract). A decrease in m_3_G/TMG RNAs at the DNA lesions was observed in the majority of irradiated cells, similarly with m^6^A RNA recruitment to DNA lesions. According to these results, we surmise that trimethylguanosine, frequently observed in snRNAs and snoRNAs, is affected by UV irradiation, which appears to be a later step in the DNA damage response.

Additionally, we observed that genome injury did not influence the level of the SC35 factor that is responsible for the splicing of pre-mRNA (Figure 7c; https://www.ncbi.nlm.nih.gov/gene/6427). Other regulatory components of RNA processing, including NOLC1 and SMD1, also were not affected by laser microirradiation (Supplementary Figure S2a,b). NOLC1, known as a nucleolar and coiled-body phosphoprotein 1 (also known as Nopp140), is a chaperone for the transcription and processing of rRNA, including rRNA assembly into small and large ribosomal subunits [52]. Moreover, NOLC1 overexpression is associated with an increased number of 53BP1 DNA damage foci [53]. Similarly to Treacle (TCOF) [54], NOLC1 (Supplementary Figure S2a) was not recruited to UVA-induced CPDs-positive sites, which are well-known not to be recognized by the 53BP1-dependent DNA repair pathway. Additionally, splicing factor SMD1 also did not recognize the UVA-damaged chromatin. This protein contributes to miRNA-mediated gene silencing through a function that is independent of its role in pre-mRNA splicing [55]. These data showed that a pronounced increase in m^6^A RNAs at DNA lesions is unique compared to other regulatory components of RNA biology.

## 4. Discussion

It is generally accepted that numerous DNA repair pathways regulate DNA repair processes, which are in high demand due to the variety of DNA lesions induced by radiation or DNA-damaging agents. However, it is likely that the DNA repair processes and/or, at least, the kinetics of the repair factors, differ in normal and tumor cells [56]. In malignant cells, non-physiological kinetics of repair factors binding to DNA lesions may indicate a disorder in repair mechanisms, which increases the probability of errors in the genome.

Fundamentally, the repair of damaged DNA sequences is primarily mediated by DNA repair proteins, but epigenetic processes, including histone posttranslational modifications and posttranscriptional modifications of RNAs, also have DNA repair functions that can be additionally affected during tumorigenesis. Despite significant progress in the development and the use of biological methods, the exact functional mechanisms of these epigenetic factors in DNA damage response is not entirely understood.

Here, we show that RNA methylation is unique in irradiated genomes. We confirmed the results of Xiang et al., who showed that m^6^A RNA accumulates at UVA-damaged chromatin, and we explain that this process could be a consequence of the coregulatory function of METTL-like enzymes, or alternatively, m^6^A RNA diffuses to UVA-damaged chromatin [22] (Figure 2(ca’–d’,d,e)). In detail, we showed that after laser microirradiation, nucleoplasmic and relatively high cytoplasmic fractions of m^6^A RNAs immediately diffuse to DNA lesions (Figure 2d,e, and Graphical Abstract). However, relatively stable levels of METTL3 and/or METTL14 enzymes were observed before and after laser light exposure (Figure 5a–c). Due to the presence of a very high level of METTL-like enzymes in micro-irradiated chromatin, this observation does not negate the hypothesis of METTL-like activity at the DNA lesions, abundant on m^6^A RNAs. In the case of the enzymatic regulation of m^6^A RNAs at CPD sites, we additionally observed the high level of the METTL16 enzyme in 10% of microirradiated cells. In these cells, we detected pronounced recruitment of the METTL16 protein to DNA lesions (Figure 5c, lower panel). From the view of RNA function, it is known that METTL16 mediates m^6^A in U6 small nuclear RNAs, as explained by Pendleton et al., who published results showing that METTL16 is a conserved U6 snRNA methyltransferase that additionally regulates SAM homeostasis [20]. Here, we document that METTL16 functions in a later step of the DNA damage response, as indicated by its level increasing 20–30 min after local laser microirradiation (Figure 5c, lower panel; Graphical Abstract). Conversely to this observation, Xiang et al. showed METTL3 as a major methyltransferase responsible for the fast accumulation of m^6^A mRNA to DNA lesions. Moreover, these authors documented that this process was accompanied by a rapid m^6^A RNA demethylation by the FTO enzyme. According to Xiang et al., this demethylation process proceeds in the period from 2 to 4 min after microirradiation, and depleting METTL3 causes a delay in the repair of the CPD sites that were significantly induced by UVA light [22]. Here, we also confirmed that m^6^A RNA appears at DNA lesions only in the presence of CPDs (Figure 4c,d). This finding was also indirectly confirmed by the results showing the effect of the Suv4-20h1/h2 inhibitor suppressing H4K20 dimethylation and subsequent binding of the 53BP1 protein to this histone mark. This epigenetic mechanism is highly specific for damaged chromatin, recognized by NHEJ repair proteins. In this regard, we observed that the Suv4-20h1/h2 inhibitor had no effect on m^6^A RNA accumulation at DNA lesions. Therefore, it seems evident that m^6^A RNAs do not play a role in the NHEJ repair pathway. However, depletion of Suv39h1/h2 enzymes (regulating H3K9me3, playing a role in NHEJ) weakens the accumulation of m^6^A RNAs at microirradiated chromatin (Figure 3(aa’,b’)). This nuclear event was likely caused by overall genome instability induced by Suv39h1/h2 deficiency that causes H3K9me3 depletion, and it is accompanied by DNA hypomethylation. In contrast to previous work [57], in Suv39h1/h2-depleted cells, we did not observe significant locus-specific hypomethylation. One of the explanations is that mutant cells are characterized by a reduced DNA methyltransferase 1 (DNMT1) level (Figure 6e,f). DNMT1 is a significant maintenance DNA methyltransferase [58]. Moreover, Suv39h1 interacts with DNMT1 in order to create a complex with additional components of the NER pathway, including PCNA and the HP1 protein [54,59]. Thus, described proteins and epigenetic factors can be considered, with a high probability, as the main epigenetic factors of the NER pathway.

Interestingly, we additionally noticed new RNA-specific phenomena of DNA damage repair. Thus, our suggestion of an epigenetic mechanism is the following: When METTL16 methylates snRNA, the subsequent process is associated with depletion of m_3_G/TMG in RNAs. This epigenetic regulation appears in an interval 20 min after local laser microirradiation when m^6^A RNAs disappear from CPD-positive sites (Figure 7(ba’,b’); Graphical abstract). Due to the fact that microirradiation did not affect the level of the phosphorylated RNA pol II [60] and because inhibitors of RNA pol I and RNA pol II did not change the accumulation of m^6^A RNA at the DNA lesions, it seems possible that epitranscriptome features related to mRNA and rRNA are to some extent resistant to UVA-irradiation. This claim is also linked to mRNA splicing because we did not observe radiation-induced changes in the levels of splicing factors, including SC-35 and SMD1 (Figure 7c, Supplementary Figure S2). Here, we only observe radiation-induced changes in factors regulating non-coding RNAs; thus, based on these results, we assume that non-coding RNAs, and especially small RNAs (snRNAs/snoRNAs) methylated at the 6 adenosine position, but not methylated mRNA or rRNA, are affected by UVA radiation.

## 5. Conclusions

Here, we suggest that the following are changes in the epitranscriptome that accompany the NER mechanism: The pronounced methylation of N^6^-adenosine in RNA is an early step of the DNA damage response. Such methylated RNAs diffuse to CPD sites, characterized by a high level of repair factors, including DNMT1 (interacting with Suv39h1, the H3K9-methyltransferase) [61], and HP1β that binds to H3K9me3 as well as playing a role in NER [54,62]. In a later step of this repair pathway, the METTL16 enzyme methylates the small RNAs (both snRNAs and snoRNAs) in the vicinity of the DNA lesions, and moreover, m^1^A RNA level is reduced in UV-irradiated cells (Figure 7a). The change in the m^1^A RNA level can be related to mRNA, t-RNA, non-coding RNAs, as well as mitochondrial genes [63]. Mentioned epigenetic events are followed by the depletion of m_3_G/TMG in a cap structure of snRNA/snoRNAs (Figure 7b). Moreover, it is well-known that U3 and U8 snoRNA localizes to Cajal bodies (CBs) during the biogenesis of small nuclear ribonucleoprotein particles (RNP), which is linked to the function of the m_3_G-cap structure [64]. Recently, we also showed that a well-known component of CBs, coilin, is pronouncedly recruited to UVA-induced DNA lesions [65]; thus, the described mechanism is significantly linked not only to splicing but also to the DNA repair processes. For an additional explanation, it is well-known that m_3_G/TMG-capped U snRNA forms a complex between snRNP and Sm proteins in the cytoplasm, which is followed by its entry into the nucleus. Importantly, the m_3_G-capped snRNP regulates pre-mRNA splicing as a part of the spliceosome [66]. However, here, local laser microirradiation does not affect the nuclear distribution pattern and the levels of splicing factors studied at the microirradiation-induced DNA lesions (Figure 7c and Supplementary Figure S2b).

## Supplementary Materials

The following are available online at https://www.mdpi.com/2073-4409/9/2/360/s1, Figure S1: The levels of proteins regulating m^6^A RNA and DNA damage response. Panels are showing the quantification of Western blot data from Figure 6e,f; Figure S2: The levels of NOCL1 (red) and SMD1 (red) factors were not changed when m_3_G/TMG was reduced by local laser microirradiation. a, The NOLC1 protein was not recruited to DNA lesions. b, the level of the SMD1 protein was identical before and after local laser microirradiation.

## Abbreviations

3′-UTRs	3′-untranslated regions
5hmC	5-hydroxymethylcytosine
5mC	5-methylcytosine
ac^4^C	N4-acetylcytidine
DAPI	4′,6-diamidino-2-phenylindole
DDR	DNA damage response
dn	double null
DNMT1	DNA methyltransferase 1
FTO	obesity-associated factor
H3K9me1/me2/me3	mono-methylated/di-methylated/tri-methylated histones 3 on lysine 9
hm^5^C	5-hydroxymethylcytidine
HMTs	histone methyltransferases
m^1^A	N^1^-*methyladenosine*
m3G/TMG	2,2,7-methylguanosine
m_3_G/TMG	2,2,7-methylguanosin
m^5^C	5-methylcytidine
m^6^A	N^6^-adenosine methylation
MEFs	mouse embryonic fibroblasts
METTL14	N^6^-adenosine-methyltransferase 14
METTL16	N^6^-adenosine-methyltransferase 16
METTL3	N^6^-adenosine-methyltransferase 3
MMU	mouse chromosome
ncRNA	noncoding RNA
NER	nucleotide excision repair mechanism
NHEJ	nonhomologous end-joining
PBS	phosphate buffered saline
PCNA	proliferating cell nuclear antigen
SAM	S-adenosylmethionine
snRNA, snoRNA, small RNAs	small RNAs
STED	stimulated emission depletion
TCOF	Treacle
UV light	ultraviolet light
WLL	white light laser
wt	wild type
γH2AX	phosphorylated histone H2AX
