# Correction: Svobodova et al. N^6^-Adenosine Methylation in RNA and a Reduced m_3_G/TMG Level in Non-Coding RNAs Appear at Microirradiation-Induced DNA Lesions. *Cells* 2020, *9*, 360

**DOI:** 10.3390/cells13211817

**Published:** 2024-11-04

**Authors:** Alena Svobodová Kovaříková, Lenka Stixová, Aleš Kovařík, Denisa Komůrková, Soňa Legartová, Paolo Fagherazzi, Eva Bártová

**Affiliations:** 1 Institute of Biophysics of the Czech Academy of Sciences, Královopolská 135, 612 00 Brno, Czech Republic; aluskakovarikova@centrum.cz (A.S.K.); lenka@ibp.cz (L.S.); kovarik@ibp.cz (A.K.); komurkova@ibp.cz (D.K.); legartova@ibp.cz (S.L.);; 2 Department of Experimental Biology, Faculty of Science, Masaryk University, Kamenice 753/5, 625 00 Brno, Czech Republic

## Error in Affiliation

The affiliation number 1, “Institute of Biophysics of the Czech Academy of Sciences, Královopolská 135, 612 65 Brno, Czech Republic” changed its zip code to 612 00. This affiliation has been updated.

## Error in Figure

In the original publication [1], there was a mistake in Figure 6 as published. Due to a technical error, Figure 6f showed identical fragments of METTL16 and histone H3 to those documented in Figure 6e. The METTL16 and histone H3 bands have been replaced in Figure 6f. The corrected Figure 6 appears below.

The authors state that the scientific conclusions are unaffected. This correction was approved by the Academic Editor. The original publication has also been updated.

## Figures and Tables

**Figure 6:**
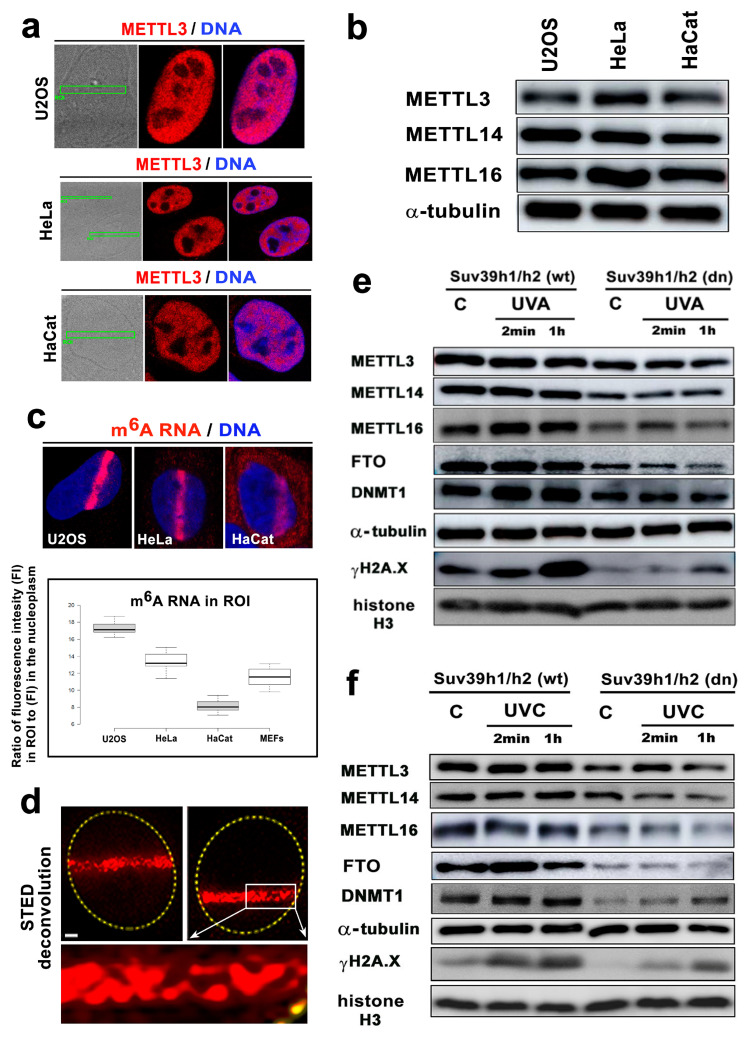
The level of m^6^A RNAs in microirradiated chromatin was the highest in the U2OS osteosarcoma cells. (**a**) METTL3 enzyme was not changed in the microirradiated U2OS and HeLa tumor cells or the HaCaT keratinocytes. (**b**) Western blotting also showed that the levels of METTL3, METTL14, and METTL16 in U2OS, HeLa, and HaCaT cells were almost identical. (**c**) In comparison to the other cell types, U2OS cells were characterized by the most pronounced accumulation of m^6^A RNAs at DNA lesions. In HaCaT cells, the level of m^6^A RNAs at the CPD sites was the lowest compared to that of the other cell types studied (see box plot). Scale bars in panels a-c represent 8 µm. (**d**) STED microscopy showed the focal distribution of the m^6^A RNAs in the microirradiated regions. Scale bars represent 2 µm. Western blot analysis of the levels of METTL3, METTL14, METTL16, FTO, DNMT1, and γH2AX in Suv39h1/h2 wt and Suv39h1/h2 dn nonirradiated cells and cells exposed to (**e**) a UVA lamp and (**f**) a UVC lamp. Protein levels were normalized to the levels of α-tubulin, and the level of histone proteins was normalized to total histone H3.
